# HTLV Deregulation of the NF-κB Pathway: An Update on Tax and Antisense Proteins Role

**DOI:** 10.3389/fmicb.2018.00285

**Published:** 2018-02-21

**Authors:** Stefania Fochi, Simona Mutascio, Umberto Bertazzoni, Donato Zipeto, Maria G. Romanelli

**Affiliations:** Department of Neurosciences, Biomedicine and Movement Sciences, University of Verona, Verona, Italy

**Keywords:** HTLV, NF-κB, Tax, HBZ, APH-2, adult T-cell leukemia, cell proliferation, apoptosis

## Abstract

Human T-cell lymphotropic virus type 1 (HTLV-1) is the causative agent of adult T-cell leukemia (ATL), an aggressive CD4^+^/CD25^+^ T-cell malignancy and of a severe neurodegenerative disease, HTLV-1 associated myelopathy/tropical spastic paraparesis (HAM/TSP). The chronic activation or deregulation of the canonical and non-canonical nuclear factor kappa B (NF-κB) pathways play a crucial role in tumorigenesis. The HTLV-1 Tax-1 oncoprotein is a potent activator of the NF-κB transcription factors and the NF-κB response is required for promoting the development of HTLV-1 transformed cell lines. The homologous retrovirus HTLV-2, which also expresses a Tax-2 transforming protein, is not associated with ATL. In this review, we provide an updated synopsis of the role of Tax-1 in the deregulation of the NF-κB pathway, highlighting the differences with the homologous Tax-2. Special emphasis is directed toward the understanding of the molecular mechanisms involved in NF-κB activation resulting from Tax interaction with host factors affecting several cellular processes, such as cell cycle, apoptosis, senescence, cell proliferation, autophagy, and post-translational modifications. We also discuss the current knowledge on the role of the antisense viral protein HBZ in down-regulating the NF-κB activation induced by Tax, and its implication in cellular senescence. In addition, we review the recent studies on the mechanism of HBZ-mediated inhibition of NF-κB activity as compared to that exerted by the HTLV-2 antisense protein, APH-2. Finally, we discuss recent advances aimed at understanding the role exerted in the development of ATL by the perturbation of NF-κB pathway by viral regulatory proteins.

## Introduction

**Figure 1:**
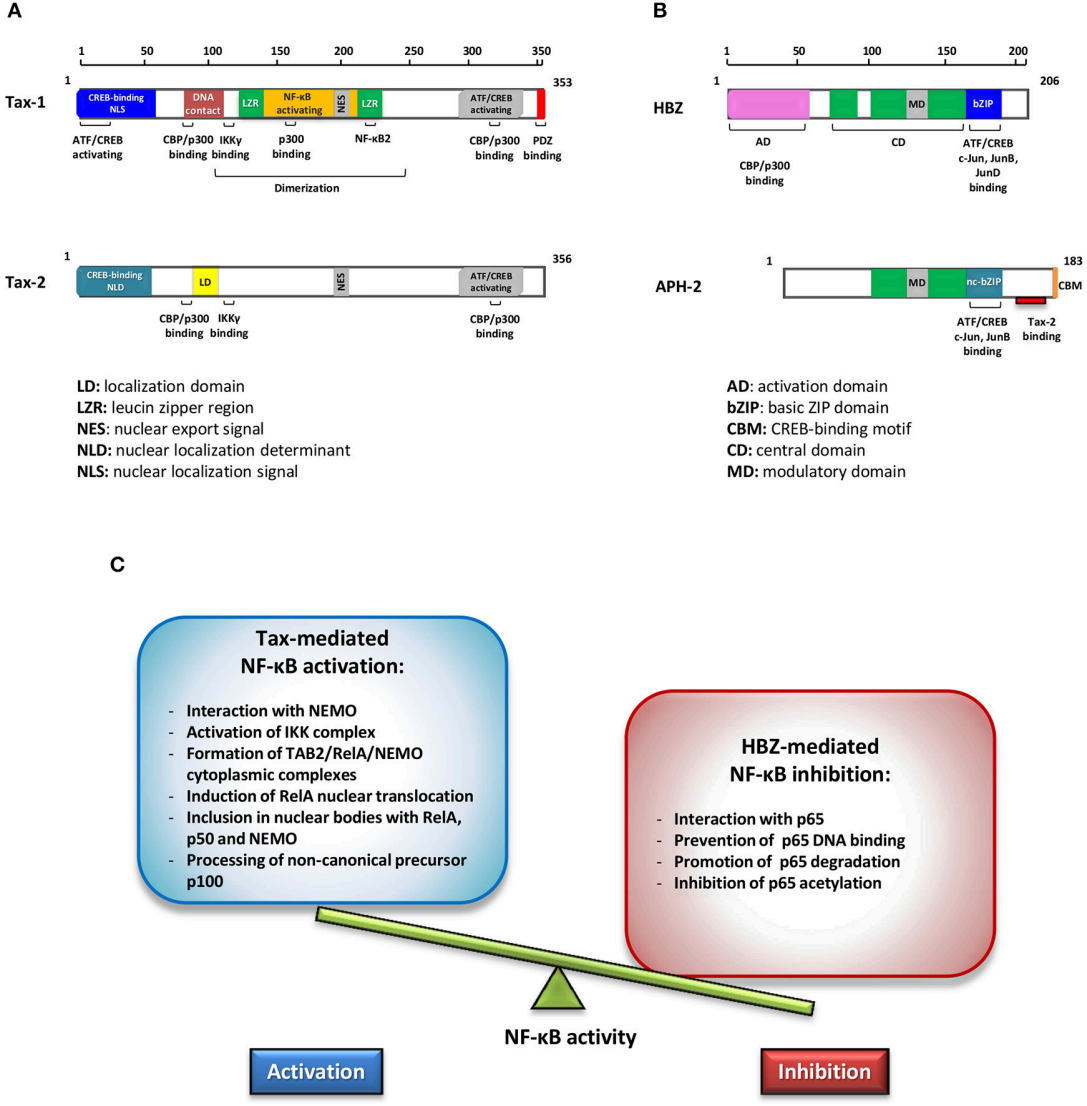
Schematic representations of Tax-1 and Tax-2 **(A)** and of HBZ and APH-2 functional domains **(B)**. Different colors highlight structural differences. **(C)** Effects of Tax and HBZ on NF-κB. Schematic representation of the opposite effects in regulating NF-κB activity exerted by Tax-1 and HBZ.

Human T-cell lymphotropic/leukemia virus type 1 (HTLV-1) is the etiological agent of adult T-cell leukemia (ATL), a malignancy of CD4^+^/CD25^+^ T cells and of a chronic inflammatory disease called HTLV-1 associated myelopathy/tropical spastic paraparesis (HAM/TSP) (Poiesz et al., 1980; Hinuma et al., 1981; Gessain et al., 1985; Gallo et al., 2017). It is estimated that at least 20 million people worldwide are infected with HTLV-1 (Gessain and Cassar, 2012; Willems et al., 2017) and approximately 5% of HTLV-1 carriers develop ATL after a latency of 20–50 years from infection (Zhang et al., 2017). HTLV-1 provirus encodes, among others, a regulatory protein, Tax and an accessory antisense strand product HTLV-1 bZip protein (HBZ), which are pivotal factors in HTLV-1 pathogenesis (Yasuma et al., 2016). Tax is a transcriptional activator of the viral long terminal repeat (LTR) with the capability to unsettle several cellular signal transduction pathways. HBZ is an inhibitor of 5′ LTR Tax-1 transactivation and is required for viral persistence (Barbeau et al., 2013). HBZ is a potent viral oncoprotein which plays an important role in deregulating several cellular processes in concerted action with Tax, affecting cell proliferation, apoptosis, autophagy, and immune escape (Zhao, 2016). Both these viral regulatory proteins promote T-cell proliferation. However, the exact mechanism underlying their role in inducing cell proliferation is still not clearly understood. The genetically related HTLV type 2 virus, although its association with ATL has not been established, encodes a homolog Tax-2 regulatory protein that induces T-cell proliferation *in vitro* and an antisense protein, named antisense protein HTLV-2 (APH-2) that, unlike HBZ, is dispensable for HTLV-2 infection and persistence (Yin et al., 2012). Their structural properties are shown in Figures 1A,B. Comparative studies between HTLV-1 and HTLV-2 have contributed to highlight differences in the virus-host interaction that may have key roles in tumorigenesis (Higuchi and Fujii, 2009; Bertazzoni et al., 2011; Romanelli et al., 2013).

Persistent activation of NF-κB by Tax is a key event for the T-cell transformation and development of ATL (Qu and Xiao, 2011; Zhang et al., 2017). Accumulating evidence indicates that the HTLVs have evolved specific strategies mediated by Tax and antisense proteins to deregulate NF-κB signaling pathways. While HBZ is consistently expressed in all ATL cells, Tax is not expressed in approximately 60% of them, even though the HTLV-1 proviral genome is integrated and NF-κB is constitutively activated (Zhao, 2016). This suggests that additional factors contribute to sustain the persistent activation of NF-κB, in the absence of Tax, in ATL cells (Matsuoka and Jeang, 2007). The alteration of the NF-κB signaling pathway could also be involved in the inflammatory state observed in HAM/TSP (Peloponese et al., 2006). An interesting aspect of Tax and HBZ functions is their opposite effect on the regulation of cellular signaling pathways (Zhao and Matsuoka, 2012; Ma et al., 2016) as further discussed here.

In this review, we summarize the recent advances in understanding the molecular mechanisms involved in NF-κB deregulation, mediated by Tax and antisense proteins, through the interaction with host factors and their roles in cell survival and proliferation.

## Tax-mediated NF-κB activation

Two distinct pathways lead to NF-κB activation, known as the canonical and the non-canonical pathways that involve different upstream, intermediate, and effector factors. A common step of both pathways is the activation of a complex that contains a serine–specific IκB kinase (IKK) composed by two catalytic kinase subunits, IKKα and IKKβ, and the regulatory non-enzymatic scaffold protein NEMO (known as IKKγ). In the canonical pathway, adaptor proteins (TRAFs) are recruited to the cytoplasmic domain of the cell membrane tumor necrosis factor receptor (TNF-R) and activate the IKK complex thus inducing the phosphorylation of IκB inhibitor and the seclusion of NF-κB precursors within the cytoplasm (Sun, 2017). This phenomenon leads to IκB degradation and nuclear translocation of the p50/RelA transcriptional effectors. At variance with the canonical pathway, the non-canonical one involves an IKK complex that does not contain NEMO, but two IKKα subunits. The NF-κB-inducing kinase (NIK) activates the IKK complex, leading to p100 processing and the final release in the nucleus of p52/RelB active heterodimer (Durand and Baldwin, 2017).

Based on the study of the molecular mechanisms of NF-κB activation driven by Tax-1, two relevant aspects emerged: the recruitment of Tax in cellular protein complexes (Bertazzoni et al., 2011; Qu and Xiao, 2011) and their post-translational modifications (Lavorgna and Harhaj, 2014). Studies comparing Tax-1 and Tax-2 have highlighted relevant differences in their activation of the NF-κB pathway as a result of protein interaction: both proteins activate the classical pathway, but only Tax-1 activates the non-canonical one; Tax-1, unlike Tax-2, triggers the activation of the non-canonical pathway recruiting NEMO and IKKα to p100, promoting the processing of p100 to p52 (Xiao et al., 2001; Higuchi et al., 2007; Shoji et al., 2009); both Tax proteins interact with TAB2 and NEMO/IKKγ stimulating the translocation of the p50/RelA heterodimers into the nucleus, but only Tax-1 interacts with TRAF6, an E3 ligase that triggers the ubiquitination and activation of the downstream NF-κB signaling cascade (Avesani et al., 2010; Journo et al., 2013). Furthermore, only Tax-1 interacts with the p52/p100 and RelB factors of the non-canonical pathway, inducing the expression of OX40L, a T-cell co-stimulatory molecule of the tumor necrosis factor family implicated in the adaptive immunity (Motai et al., 2016).

We have recently shown that Tax-1 and Tax-2 form complexes with two homologous non-canonical IκB kinases, IKKε and TBK1, which are not component of IKK complexes, but are implicated in the activation of NF-κB, STAT3 and induction of IFNα (Shen and Hahn, 2011; Diani et al., 2015). An additional study demonstrating the presence of Tax and TBK1 in lipid raft microdomains along with canonical IκB supports the role of Tax-1 as a promoter of the molecular crosstalk between the canonical IKKs and additional signaling pathways involved in cell survival and proliferation (Zhang et al., 2016). Interestingly, it has also been reported that Tax-1 forms complexes with the ubiquitin-conjugating enzyme Ubc13, NEMO, Tax1 binding protein1 (TAX1BP) and NRP/Optineurin in the membrane lipid rafts microdomain. In these complexes, the cell adhesion molecule 1 (CADM1) acts as a molecular scaffold recruiting Tax-1 (Pujari et al., 2015). This interaction contributes to the activation of the IKK complex and the inactivation of the NF-κB negative regulator A20 enzyme, thus maintaining a persistent NF-κB activation. An additional consequence of the Tax reorganization of the component of the lipid raft is the deregulation of autophagy. Tax-1, in fact, participates to the connection of the IKK complex to the autophagy molecular complexes by interacting directly with Beclin1 and PI3KC3 and contributing to the assembly of autophagosomes (Ren et al., 2012, 2015; Chen et al., 2015). Tax-1 induction of NF-κB also increases the expression of inhibitors of apoptosis, such as the anti-apoptotic *c-Flip* gene, and of genes involved in cell cycle progression, including cyclin D2, cyclin E, E2F1, CDK2, CDK4, and CDK6 (Wang et al., 2014; Bangham and Matsuoka, 2017; Karimi et al., 2017).

It has been recently reported that Tax-activation of NF-κB can be suppressed by host factors. Among them, the transcriptional regulator of the major histocompatibility complex class II (CIITA) impairs the nuclear translocation of RelA and directly interacts with Tax-1/RelA in nuclear bodies, preventing Tax-1 mediated activation of NF-κB-responsive promoters (Forlani et al., 2013, 2016). In addition, the apoptotic regulator Bcl-3 has been demonstrated to inhibit RelA nuclear translocation and its DNA binding activity, resulting in a downregulation of Tax-induced NF-κB activation (Wang et al., 2013). The decrease in Tax-NF-κB activation could also be due to Tax proteasomal degradation induced by host factor interaction (Lavorgna and Harhaj, 2014). Tax-1 interaction with the molecular chaperone HSP90 was shown to protect Tax from proteasomal degradation (Gao and Harhaj, 2013), whereas the interaction with PDLIM2 (PDZ-LIM domain-containing protein) within the nuclear matrix induces its polyubiquitination-mediated proteasomal degradation (Yan et al., 2009; Fu et al., 2010). Furthermore, two tumor suppressor genes, MDFIC and MDF, have been recently identified as Tax-1 interactors that alter its subcellular distribution and stability, reducing Tax-dependent activation of NF-κB (Kusano et al., 2015).

The second major mechanism required for Tax-1 and Tax-2 NF-κB activation is the process of post-translational modification, which includes ubiquitination, SUMOylation and phosphorylation. It is well established that Tax phosphorylation is required for its nuclear translocation and stabilization in the nuclear bodies containing RelA (Bex et al., 1999; Turci et al., 2006). The requirements of ubiquitination and SUMOylation are more complex to define. Both the E2 enzyme Ubc13 and the E3 Ring Finger Protein 8 (RNF8) promote Tax K63-linked polyubiquitination and are essential for the activation of the IKK complex (Shembade et al., 2007; Ho et al., 2015). Other proteins, including E3 ubiquitin ligases, TRAF2, 5, or 6, can potentiate Tax polyubiquitination (Yu et al., 2008). SUMOylated Tax has been demonstrated to bind p300, RelA and NEMO in nuclear bodies (Nasr et al., 2006). In addition, SUMOylation of Tax may be involved in the regulation of Tax stability and NF-κB pathway activation (Kfoury et al., 2011). We have described that SUMOylation and ubiquitination influence Tax proteins intracellular localization, as well as the interaction with NF-κB factors and their transactivating activity (Turci et al., 2012). However, the role of Tax SUMOylation in NF-κB activation remains controversial, given that Tax-induced IKK activation has been shown to correlate with the level of Tax ubiquitination, but not with Tax SUMOylation (Bonnet et al., 2012; Pène et al., 2014). A recent study suggests that Tax itself may function as an ubiquitin E3 ligase that, in association with the ubiquitin-conjugating enzyme E2, catalyzes the assembly of mixed polyUb chains (Wang et al., 2016). However, a more recent study does not attribute to Tax an E3 ligase activity, while suggesting that multivalent interactions between NEMO proteins and Ub-chains can lead to the formation of a macromolecular Taxisome and consequently to the activation of the IKK complex (Shibata et al., 2017).

An additional mechanism that operates within the cells to maintain the NF-κB activation induced by Tax-1 is the positive feedback loop derived by NF-κB target genes. A recent report describes that the over-expression of the early growth response protein 1 (EGR1) induced by Tax-1 activation of NF-κB, results in the stabilization of EGR1 by direct interaction with Tax and nuclear translocation of p65, enhancing NF-κB activation (Huang et al., 2017). A similar positive loop is fostered by the overexpression of the interleukin receptor IL-17RB. Tax-1 promotes the expression of IL-17RB by NF-κB activation and establishes an IL-17RB-NF-κB feed-forward autocrine loop that maintains persistent NF-κB activation (Lavorgna et al., 2014).

## Tax and HBZ interplay on NF-κB deregulation

HBZ can promote viral latency by antagonizing many of the activities mediated by Tax. HBZ inhibits the activation of the HTLV-1 5′ LTR preventing the formation of the Tax transactivation complex (Gaudray et al., 2002; Clerc et al., 2008). The activation of the classical NF-κB pathway by Tax is inhibited selectively by HBZ expression (Zhao et al., 2009; Wurm et al., 2012). This inhibition is connected to the following properties of HBZ as shown in Figure 1C: (a) the interaction with p65; (b) the inhibition of p65 DNA binding; (c) the enhanced degradation of p65 through PDLIM2 E3 ubiquitin ligase; (d) the reduction of p65 acetylation. All these processes result in the reduction of the expression of several NF-κB target genes. A typical example is the *cyclin D1* promoter gene, an essential regulator of the G1/S phase transition of the cell cycle that is overexpressed by Tax-mediated NF-κB activation, while it is downregulated by HBZ interaction with p65 (Ma et al., 2017).

The HBZ inhibition of NF-κB has been proposed to be a critical step in the oligoclonal expansion of HTLV-1-infected cells by downregulating the senescence process (Giam and Semmes, 2016). NF-κB hyper-activation induced by Tax leads to the over-expression of the cyclin-dependent kinase inhibitors, p21 and p27, thus promoting an arrest of cell proliferation that triggers senescence. The proposed model envisages that in HTLV-1 infected cells, in which the p21/p27 functions is impaired, the HBZ downregulation of NF-κB may contrast the senescence induced by Tax hence promoting the expansion of the infected cells (Kuo and Giam, 2006; Zhang et al., 2009; Zhi et al., 2011).

In contrast to HBZ, the HTLV-2 homolog protein APH-2 is dispensable for HTLV infection and persistence and does not promote T-cell proliferation *in vitro* (Yin et al., 2012; Barbeau et al., 2013). In addition, APH-2 expression correlates with the proviral load in HTLV-2 infected subjects and, contrary to HBZ, does not promote lymphocytosis (Saito et al., 2009; Douceron et al., 2012). Of note, HBZ and APH-2 also diverge in the interaction with Tax, since HBZ does not bind Tax-1, whereas Tax-2 interacts with APH-2 (Marban et al., 2012). A recent study has shown that despite HBZ and APH-2 interact with p65/RelA and repress its transactivation activity in transfected cells, they diverge in the induction of p65 degradation since this is not detected in the presence of APH-2 (Panfil et al., 2016). This different effect suggests that the two proteins may adopt different mechanisms to interfere with NF-κB activation. The differences between regulatory proteins of HTLV-1 and HTLV-2 in deregulating NF-κB are outlined in Table 1.

**Table 1:** Comparative effects of HTLV regulatory proteins on NF-κB pathways.

	**Tax-1**	**Tax-2**	**References**
Canonical NF-κB transactivation	+	+	Sun et al., 1994
Non-canonical NF-κB transactivation	+	−	Higuchi et al., 2007
NF-κB transactivation (lipid raft translocation of IKK)	+	−	Huang et al., 2009
Interaction with p100/p52	+	−	Shoji et al., 2009
Interaction with p65	+	+	Zhao et al., 2009; Panfil et al., 2016
	**HBZ**	**APH-2**	**References**
Canonical NF-κB inhibition	+	+	Zhao et al., 2009; Panfil et al., 2016
Non-canonical NF-κB inhibition	−	nd	Zhao et al., 2009
Interaction with p65	+	+	Zhao et al., 2009; Panfil et al., 2016
Inhibition of Tax-mediated transactivation of NF-κB	+	nd	Zhao et al., 2009
Binding to Tax	−	+	Marban et al., 2012
Inhibition of p65 DNA binding capacity	+	nd	Zhao et al., 2009
p65 degradation	+	−	Panfil et al., 2016
Inhibition of p65 acetylation	+	nd	Wurm et al., 2012

*nd, Not determined*.

## Role of tax and HBZ in ATL development

The opposite functions of Tax and HBZ in the regulation of signaling pathways and their effects in survival and proliferation appear as relevant steps during HTLV-1 cellular transformation and tumorigenesis (Giam and Semmes, 2016; Bangham and Matsuoka, 2017; Zhang et al., 2017). The absence of Tax expression in the late stages of the infection is linked to *tax* gene mutations and DNA methylation of the 5′ LTR provirus (Furukawa et al., 2001; Koiwa et al., 2002). On the opposite, the 3′ LTR negative strand remains intact and non-methylated, allowing HBZ to be systematically expressed in ATL cells (Taniguchi et al., 2005; Miyazaki et al., 2007). Unlike HBZ, Tax-1 is highly immunogenic and its inactivation may represent a fundamental strategy to evade the host immune system, a critical step in ATL development (Kogure and Kataoka, 2017). HBZ, like Tax-1, deregulates cell proliferation by targeting key factors implicated in cell survival. HBZ, in fact, binds to ATF3/p53 complexes and inhibits the p53 expression induced by ATF3, thus promoting ATL cells proliferation (Hagiya et al., 2011). HBZ also induces the expression of the anti-apoptotic genes *BCL2* and *Flip*, interacting with C/EBPα and deregulating the C/EBP signaling (Zhao et al., 2013). Both Tax-1 and HBZ are involved in the inhibition of the tumor suppressor p53. In particular, Tax inhibits p53 activity through the p65 subunit of NF-κB or by sequestering p300/CBP from p53 (Ariumi et al., 2000; Karimi et al., 2017). Recent studies revealed that HBZ, by binding p300/CBP, inhibits p53 acetylation and decreases the p53 activity (Wright et al., 2016).

The selectivity of HBZ in inhibiting the classical NF-κB pathway opens an interesting area of investigation on the role of the non-canonical NF-κB pathway in tumorigenesis. During ATL development, HBZ might downmodulate the classical NF-κB pathway more efficiently when Tax expression is silenced, leading to predominant activation of the alternative pathway (Zhao et al., 2009). It has also been demonstrated that freshly isolated ATL cells display high expression levels of NIK, persistent phosphorylation of IκBα, aberrant processing of p52, and nuclear translocation of p50, p52, and RelB, despite the absence of Tax-1 expression (Chan and Greene, 2012).

Genetic and epigenetic alterations, including miRNAs expression profile, have been intensively investigated in the genome of ATL patients (Yeung et al., 2008; Bellon et al., 2009; Yamagishi and Watanabe, 2012; Watanabe, 2017). It has been proposed that the genomic instability may derive from Tax inhibition of DNA double-strand break repair and induction of micronuclei formation. ATL cells are characterized by frequent gain-of-function alterations of genes involved in the T-cell receptor/NF-κB signaling pathway, such as PLCG1, PKCB, and CARD11 or loss-of-function mutations in upstream factors, such as TRAF3 (Cook et al., 2017; Kogure and Kataoka, 2017). Mutations or intragenic deletions of these genes result in NF-κB induction in the absence of Tax-1. A progressive epigenetic downregulation of *miR-31* has been demonstrated in ATL (Fujikawa et al., 2016). Of note, miR-31 negatively regulates the expression of NIK and miR-31 loss in ATL triggers the persistent activation of NF-κB, inducing apoptosis resistance and contributing to the abnormal proliferation of cancer cells (Yamagishi et al., 2012). In addition, Fujikawa et al. (2016) showed that Tax-mediated NF-κB activation induces the over-expression of the histone-lysine methyltransferase, EZH2, leading to host epigenetic machinery deregulation. It has been proposed that EZH2 may contribute to NF-κB activation through miR-31 silencing and consequently NIK induction, in a positive feedback loop (Sasaki et al., 2011; Fujikawa et al., 2016). Genetic mutations have been also suggested to cause IL-17RB overexpression which triggers classical NF-κB activation by an autocrine-loop in a subset of Tax-negative ATL cell lines (Lavorgna et al., 2014).

## Conclusions and perspectives

HTLV-1 appears to benefit from the antagonistic functions of Tax and HBZ in the deregulation of cellular signaling pathways, resulting in the loss of control of many biological processes such as proliferation and survival of HTLV-1-infected cells. The interplay between Tax and HBZ on NF-κB regulation has a prominent role in viral persistence in ATL cells, thus contributing to leukemic transformation. The intensive studies conducted in recent years aimed at understanding the effect of Tax constitutive activation and HBZ inhibition of NF-κB have contributed to further elucidate the molecular mechanism of NF-κB activation. However, several open questions about its functional role in ATL development still need to be addressed: the exact role of the persistent NF-κB activation in ATL cells; the contribution to tumorigenesis of the alternative pathway activation; the role of the different mechanisms that are adopted by HBZ and APH-2 to interfere with NF-κB activation; the dynamic organization of lipid raft complexes in HTLV-1 infected cell. It is hoped that the application of the CRISPR/Cas9 genome editing new technique will offer a useful tool to investigate the requirement of specific interactions of Tax and HBZ with cell factors that activate the mechanisms driving to tumorigenesis.

## Author contributions

SF, SM, and MR wrote the review. SF, SM, UB, DZ, and MR participated in the conception and design of the review. All authors read and approved the final manuscript.

### Conflict of interest statement

The authors declare that the research was conducted in the absence of any commercial or financial relationships that could be construed as a potential conflict of interest. The reviewer JY and handling Editor declared their shared affiliation.
